# Shifting paradigms in 30,427 surgical colorectal cancer cases (2008–2023): the impact of endoscopic screening on caseload complexity and the value of minimally invasive quality

**DOI:** 10.1007/s00464-026-12818-1

**Published:** 2026-04-20

**Authors:** Zhenting Lu, Junzhe Tang, Shiqi Hu, Dakui Luo, Xinyi Wang, Xinxiang Li, Qingguo Li

**Affiliations:** 1 Department of Colorectal Surgery, Fudan University Shanghai Cancer Center, Shanghai, 200032 China; 2 Department of Oncology, Shanghai Medical College, Fudan University, Shanghai, 200032 China; 3 Department of Rheumatology, Peking Union Medical College Hospital (PUMCH), Peking Union Medical College and Chinese Academy of Medical Sciences, National Clinical Research Center for Dermatologic and Immunologic Diseases (NCRC-DID), Key Laboratory of Rheumatology and Clinical Immunology, Ministry of Education, Beijing, China

**Keywords:** Colorectal cancer, Laparoscopic surgery, Endoscopic screening, Early-onset colorectal cancer, Lymph node yield, Stage migration

## Abstract

**Background:**

While global registries report a rising incidence of early-onset colorectal cancer (EOCRC), the impact of widespread screening and endoscopic therapeutics on the case-mix of major surgical centers remains undercharacterized. We aimed to quantify the 16-year evolution of surgical demographics, pathological profiles, and quality metrics to delineate how modern practice reshapes surgical volume and outcomes.

**Methods:**

We conducted a retrospective analysis of 30,427 consecutive patients undergoing colorectal cancer (CRC) resection at Fudan University Shanghai Cancer Center from 2008 to 2023. The cohort was stratified into three eras: Era 1 (Open-predominant, 2008–2012), Era 2 (Transitional, 2013–2017), and Era 3 (Minimally Invasive-predominant, 2018–2023). Trends in demographics, tumor morphology, stage distribution, lymph node yield, and 5-year overall survival (OS) were analyzed.

**Results:**

Contrary to global incidence trends, the proportion of surgically managed EOCRC (< 50 years) declined from 24.8% in 2008 to 14.6% in 2023, while patients aged ≥ 65 years increased from 27.4 to 39.4% (*P* < 0.001). A pattern consistent with a hypothesized “endoscopic filtering effect” was observed: the proportion of surgical Stage 0–I cases contracted from 20.3 to 12.4%, while adenoma with high-grade intraepithelial neoplasia (HGIN) rose dramatically from 1.5 to 12.3%. Mean tumor diameter exhibited a “J-shaped” trajectory: decreasing from 4.20 cm in 2012 to a nadir of 3.80 cm in 2014, before rebounding to 4.20 cm by 2023. Surgically, laparoscopy adoption surged from 0.2 to 64.5%. This transition was accompanied by a steady increase in mean lymph node yield, rising from 15.4 in Era 1 to 16.9 in Era 3 (*P* < 0.001). In 2023, the mean lymph node yield in the laparoscopic group (16.3 ± 6.8) was numerically superior to the open surgery group (15.6 ± 7.7; *P* = 0.067). Regarding prognosis, 5-year OS peaked during the transitional period (Era 2: 75.9%). Although OS declined in Era 3 (69.5%) concurrent with the shift toward an older and more complex case-mix, outcomes remained robust and comparable to the historical baseline (Era 1: 73.0%).

**Conclusions:**

The landscape of surgical CRC has shifted toward an older population with more complex, locally advanced disease, a pattern consistent with upstream endoscopic diversion of early-stage cases. Despite this increased demographic burden, the maturation of minimally invasive techniques has maintained high-quality oncological clearance, as evidenced by improving lymph node yield and declining circumferential resection margin (CRM) positivity rates. After adjusting for age, sex, stage, tumor location, and surgical approach, survival outcomes in the most recent era were comparable to the historical baseline (HR = 1.049, 95% CI 0.976–1.128, *P* = 0.194).

**Graphical abstract:**

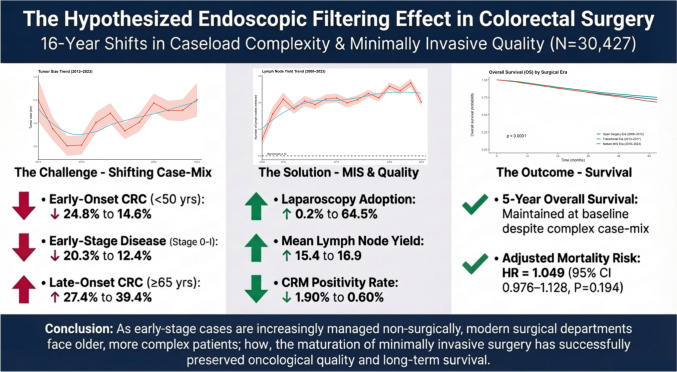

**Supplementary Information:**

The online version contains supplementary material available at 10.1007/s00464-026-12818-1.

Colorectal cancer (CRC) remains a formidable global health challenge, ranking as the third most common malignancy and the second leading cause of cancer-related mortality worldwide [1]. Contemporary epidemiology has highlighted a concerning demographic shift: while incidence rates in older adults have stabilized or declined in many developed nations due to screening, there is a global surge in early-onset colorectal cancer (EOCRC) among individuals younger than 50 years [2–5]. Consequently, international guidelines have increasingly advocated for lowering the age of screening initiation to 45 years [6–8]. Parallel to these epidemiological changes, the widespread adoption of opportunistic screening and advancements in therapeutic endoscopy—such as endoscopic submucosal dissection (ESD)—have revolutionized the management of precursor lesions (e.g., advanced adenomas) and early-stage carcinomas, allowing for organ-preserving curative treatments[9, 10].

However, a critical knowledge gap exists between population-level cancer statistics and the "real-world" case-mix observed in high-volume surgical centers [11, 12]. Most epidemiological studies report incidence based on cancer registries, which aggregate all diagnosed cases regardless of treatment modality. These data do not reflect the specific burden faced by surgical departments [12, 13]. As effective screening programs and advanced endoscopy increasingly “filter out” pre-malignant lesions and early-stage cancers (Stage 0–I) upstream, the residual cohort presenting for surgical resection is hypothesized to undergo a fundamental transformation [14, 15]. We postulate that modern surgical departments are increasingly becoming centers for "refractory" or "unscreened" populations—specifically, older patients with more complex, locally advanced disease—rather than a mirror image of general population trends.

Furthermore, the surgical management of CRC has undergone its own paradigm shift over the past two decades with the universal diffusion of minimally invasive surgery (MIS) [16, 17]. While laparoscopic surgery has been proven oncologically equivalent to open surgery in randomized controlled trials, its performance in the context of this shifting, increasingly complex patient demographic (e.g., aging population with bulkier tumors) requires real-world validation [18–20]. Specifically, whether the "learning curve" of MIS has impacted critical quality metrics, such as lymph node yield, during the transition from open to laparoscopic dominance remains a pivotal question [21–23].

In this study, we leveraged a large-scale, single-center database comprising 30,427 consecutive surgical cases over a 16-year period (2008–2023). Unlike multi-center registries that may suffer from heterogeneous data quality, this single-institution cohort offers high-granularity data to trace the continuous evolution of surgical practice. We aimed to: (1) quantify the shifts in patient demographics and tumor characteristics to test the hypothesis of an “endoscopic filtering effect”; and (2) evaluate the evolution of surgical quality and long-term survival outcomes amidst the transition to a minimally invasive standard.

## Methods

### Study design and patient selection

This single-center, retrospective cohort study was conducted at Fudan University Shanghai Cancer Center (FUSCC), a high-volume tertiary academic center in China. We reviewed the records of consecutive patients who underwent surgical resection for primary colorectal cancer between January 1, 2008, and December 31, 2023.

Inclusion criteria were: (1) pathologically confirmed colorectal adenocarcinoma or high-grade intraepithelial neoplasia (HGIN) managed by formal surgical resection. HGIN cases were included because they represent a clinically relevant surgical population whose increasing identification reflects the evolving interface between endoscopic and surgical management; a sensitivity analysis excluding these cases was performed to confirm the robustness of the main findings; and (2) primary resection with curative intent. Exclusion criteria included: (1) patients with synchronous multiple primary cancers; (2) cases with incomplete clinicopathological data preventing staging; and (3) data from the years 2006–2007 due to inconsistent electronic medical record (EMR) registration during the system’s implementation phase. The final analysis cohort comprised 30,427 patients. The study protocol was approved by the Institutional Review Board of FUSCC, and the requirement for informed consent was waived due to the retrospective nature of the study.

### Temporal stratification and data variables

To capture the evolution of surgical practice and case-mix, the 16-year study period was stratified into three distinct chronological eras based on the penetration of minimally invasive surgery (MIS) at our institution:

- **Era 1 (Open-predominant, 2008–2012):** Representing the early period with low laparoscopic adoption (*n* = 5,270).
- **Era 2 (Transitional, 2013–2017):** A period of rapid diffusion of laparoscopic techniques (*n* = 9,263).
- **Era 3 (MIS-predominant, 2018–2023):** The mature period where MIS became the standard of care (*n* = 15,894). This classification was designed primarily as a clinically intuitive temporal framework to describe the natural evolution of surgical case-mix, rather than to evaluate the independent effect of laparoscopic surgery per se. Continuous time trend analyses using calendar year as a continuous variable were additionally performed to validate findings independent of this era classification.

Clinicopathological variables extracted included patient demographics (age, sex), tumor location (right colon, left colon, rectum), surgical approach (open vs. laparoscopic), total lymph node yield, and pathological staging. Tumor location was defined as: right colon (cecum to transverse colon), left colon (splenic flexure to sigmoid colon), and rectum. Pathological staging was standardized according to the 8th edition of the American Joint Committee on Cancer (AJCC) TNM staging system.

Specific Note on Tumor Size Analysis: Data regarding maximum tumor diameter were analyzed separately. Due to a high rate of missing anthropometric tumor measurements in the EMR system prior to 2012, the analysis of tumor size dynamics was strictly restricted to the period from 2012 to 2023 to ensure data quality and statistical reliability.

### Outcome definitions and follow-up

The primary quality metric was lymph node yield. The primary survival outcome was Overall Survival (OS), defined as the time from the date of surgery to death from any cause or the date of last follow-up. Vital status was ascertained through active follow-up via telephone interviews and linkage with the Shanghai Center for Disease Control and Prevention (CDC) death registry. For the purpose of comparative analysis across eras with varying follow-up durations, survival time was censored at 60 months (5 years).

### Statistical analysis

Categorical variables were compared using the Chi-square test or Cochran–Armitage test for trend, as appropriate. Continuous variables (e.g., age, tumor size, lymph node yield) were expressed as mean ± standard deviation (SD) and compared using one-way ANOVA or the Kruskal–Wallis test. To visualize the adoption of surgical techniques and stage migration, stacked area charts were generated. Temporal trends in tumor size and lymph node yield were visualized using locally estimated scatterplot smoothing (LOESS) curves with 95% confidence intervals (CI). Survival curves were estimated using the Kaplan–Meier method and compared using the log-rank test. All statistical analyses were performed using R software (Version 4.5.1). Multivariable Cox proportional hazards regression was performed to evaluate the independent effect of surgical era on overall survival, adjusting for age, sex, TNM stage, tumor location, and surgical approach. Hazard ratios (HR) with 95% confidence intervals (CI) were reported. The proportional hazards assumption was assessed using Schoenfeld residual testing; where violations were detected, stratified Cox models were employed as sensitivity analyses. In addition to lymph node yield, circumferential resection margin (CRM) status was evaluated as a supplementary indicator of surgical quality. A sensitivity analysis excluding all cases of high-grade intraepithelial neoplasia (HGIN) was performed to assess the robustness of the main findings. Continuous time trend analyses using calendar year as a continuous variable were also conducted as an alternative to the era-based classification. A two-sided *P*-value < 0.05 was considered statistically significant.

## Results

### The “screening effect”: demographic and pathological shifts

**Fig. 1:**
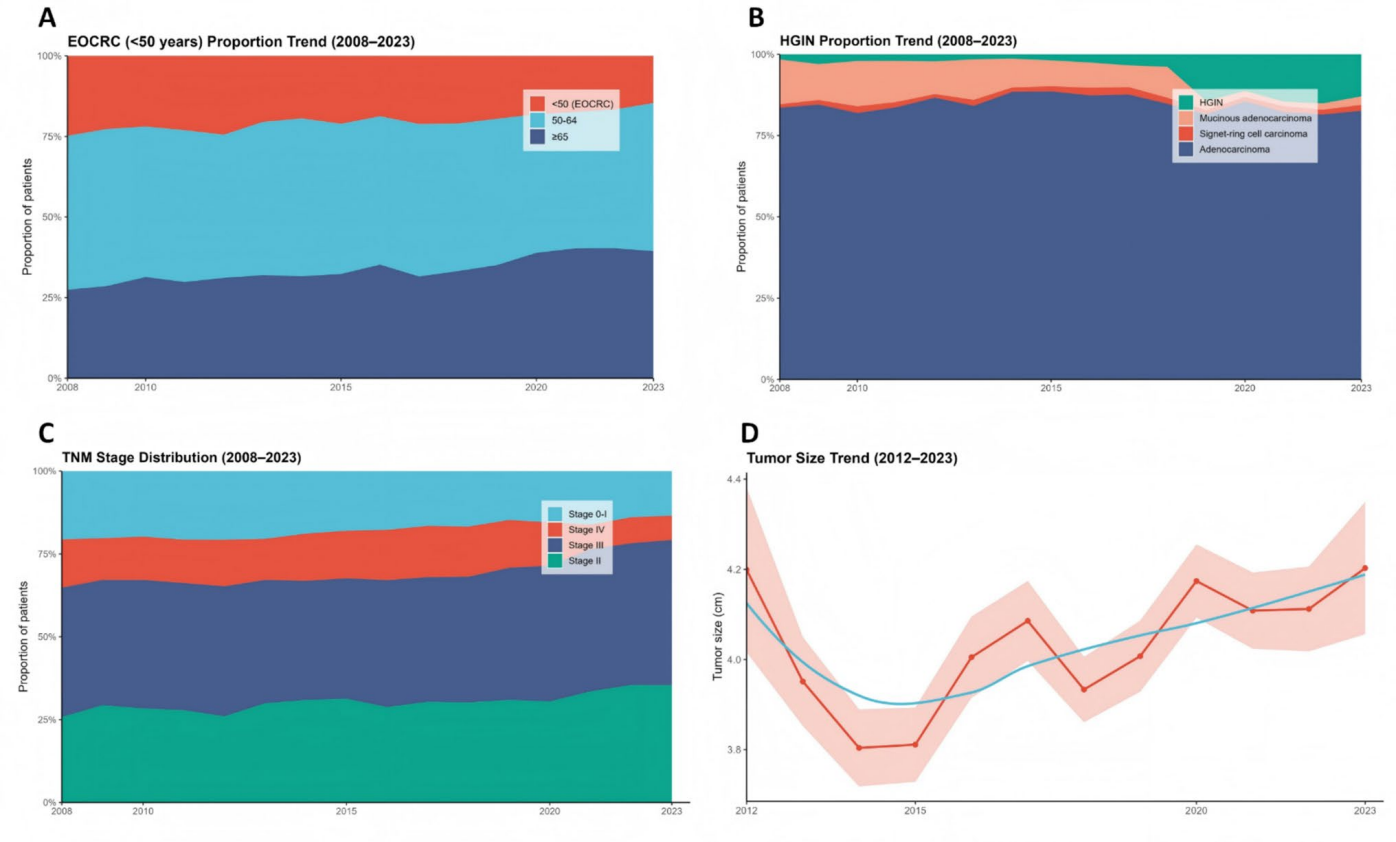
Temporal evolution of surgical caseload characteristics reflecting the “endoscopic filtering effect” (2008–2023). **A** Temporal trends in age distribution, showing a decline in the proportion of early-onset CRC (< 50 years) and a simultaneous expansion of the late-onset cohort (≥ 65 years). **B** Evolution of pathological profiles, highlighting the significant rise in the detection of adenoma with high-grade intraepithelial neoplasia (HGIN) and the decrease in mucinous adenocarcinoma. **C** Stacked area chart of TNM stage distribution, demonstrating the contraction of the Stage 0–I surgical volume (upstream filtering) and the relative expansion of Stage II and III cases. **D** Trends in mean tumor diameter (with 95% CI ribbon), exhibiting a distinct “J-shaped” trajectory: an initial decline (2012–2014) followed by a rebound (2015–2023), suggesting the successful endoscopic diversion of smaller lesions

A total of 30,427 patients were included in the final analysis. The study cohort exhibited a profound demographic shift over the 16-year period. Contrary to reports of rising early-onset colorectal cancer (EOCRC) in the general population, the proportion of surgically managed patients aged < 50 years declined substantially from 24.8% in 2008 to 14.6% in 2023. Conversely, the late-onset group (≥ 65 years) expanded from 27.4% to 39.4% (*P* < 0.001), indicating a rapid aging of the surgical caseload (Fig. 1A; Table 1).

**Table 1:** Demographic and clinicopathologic characteristics of surgically managed colorectal cancer stratified by surgical era (2008–2023)

Variable	Overall (*N* = 30,427	Era 1 (2008–2012) (*n* = 5270)	Era 2 (2013–2017) (*n* = 9263)	Era 3 (2018–2023) (*n* = 15,894)	*P* Value
Demographics					
Age, years (mean ± SD)	59.2 ± 11.9	58.1 ± 12.3	59.0 ± 11.7	59.7 ± 11.8	< 0.001
Age group, *n* (%)					< 0.001
Early-onset (< 50 years)	5988 (19.7%)	1233 (23.4%)	1871 (20.2%)	2884 (18.1%)	
Intermediate (50–64 years)	13,840 (45.5%)	2460 (46.7%)	4376 (47.2%)	7004 (44.1%)	
Late-onset (≥ 65 years)	10,599 (34.8%)	1577 (29.9%)	3016 (32.6%)	6006 (37.8%)	
Sex, *n* (%)					0.017
Male	18,312 (60.2%)	3085 (58.5%)	5570 (60.1%)	9657 (60.8%)	
Female	12,115 (39.8%)	2185 (41.5%)	3693 (39.9%)	6237 (39.2%)	
Tumor characteristics					
Tumor location, *n* (%)					< 0.001
Right colon	7169 (23.7%)	1484 (28.2%)	1930 (20.8%)	3755 (23.8%)	
Left colon	7131 (23.5%)	1041 (19.8%)	2082 (22.5%)	4008 (25.4%)	
Rectum	15,948 (52.6%)	2731 (51.9%)	5220 (56.4%)	7997 (50.7%)	
Whole/indeterminate	54 (0.2%)	11 (0.2%)	27 (0.3%)	16 (0.1%)	
Tumor size, cm (mean ± SD)	4.04 ± 2.18	4.19 ± 2.35	3.95 ± 1.97	4.08 ± 2.28	< 0.001
Pathology & stage					
Histology, *n* (%)					< 0.001
Adenocarcinoma	25,027 (84.6%)	4433 (84.3%)	8051 (87.3%)	12,543 (83.2%)	
Mucinous adenocarcinoma	1942 (6.6%)	638 (12.1%)	790 (8.6%)	514 (3.4%)	
HGIN	2107 (7.1%)	114 (2.2%)	205 (2.2%)	1788 (11.9%)	
Signet-ring cell carcinoma	491 (1.7%)	76 (1.4%)	177 (1.9%)	238 (1.6%)	
TNM stage, *n* (%)					< 0.001
Stage 0–I	4894 (17.1%)	1056 (20.4%)	1592 (18.2%)	2246 (15.3%)	
Stage II	8828 (30.8%)	1418 (27.4%)	2649 (30.2%)	4761 (32.4%)	
Stage III	11,313 (39.5%)	2000 (38.7%)	3255 (37.2%)	6058 (41.2%)	
Stage IV	3607 (12.6%)	696 (13.5%)	1264 (14.4%)	1647 (11.2%)	
Surgical quality & outcomes					
Surgical approach, *n* (%)					< 0.001
Open surgery	19,975 (65.6%)	5037 (95.6%)	6904 (74.5%)	8034 (50.5%)	
Laparoscopic surgery	10,452 (34.4%)	233 (4.4%)	2359 (25.5%)	7860 (49.5%)	
Lymph node yield (mean ± SD)	16.4 ± 7.6	15.4 ± 7.4	16.1 ± 7.5	16.9 ± 7.7	< 0.001
Adequate LN yield (≥ 12), *n* (%)	24,218 (79.6%)	3946 (74.9%)	7402 (79.9%)	12,870 (81.0%)	< 0.001
5-year overall survival (%)	73.2%	73.0%	75.9%	69.5%	< 0.001

A pattern consistent with a hypothesized :endoscopic filtering effect” was observed across pathological and staging dimensions. The detection rate of adenoma with high-grade intraepithelial neoplasia (HGIN) in the surgical department increased nearly eightfold, rising from 1.5% in 2008 to 12.3% in 2023 (Fig. 1B). This trend suggests a significant improvement in the identification and referral of precursor lesions. Simultaneously, the proportion of favorable histology, such as mucinous adenocarcinoma, decreased from 13.7 to 2.5%.

Consistent with the diversion of early lesions to non-surgical management, the proportion of surgical Stage 0–I cases contracted significantly from 20.3% in 2008 to 12.4% in 2023 (*P* < 0.001), leaving Stage II and III cases as the dominant volume (Fig. 1C). Morphologically, the mean tumor diameter exhibited a characteristic "J-shaped" trajectory (Fig. 1D). Mean tumor size initially decreased from 4.20 cm in 2012 to a nadir of 3.80 cm in 2014, likely reflecting initial screening awareness. However, this was followed by a steady rebound, reaching 4.20 cm by 2023. This paradoxical increase implies that as smaller lesions are increasingly managed endoscopically, the surgical department is progressively reserved for larger, more complex tumors.

### Evolution of surgical quality in the MIS Era

**Fig. 2:**
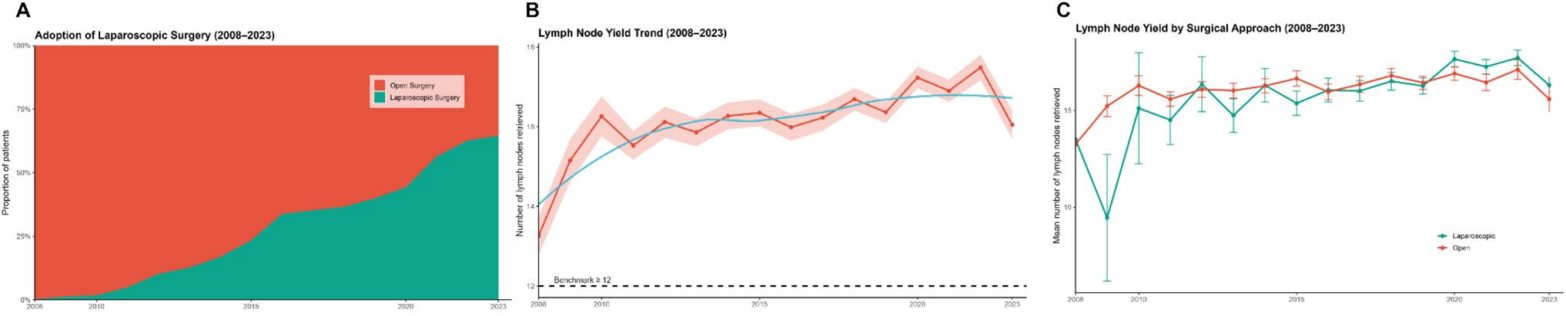
The paradigm shift to minimally invasive surgery and evolution of oncological quality metrics. **A** The adoption curve of laparoscopic surgery, showing a rapid transition from an open-surgery predominant era (< 1% in 2008) to a mature MIS era (> 60% in 2023). **B** Longitudinal trend in mean total lymph node yield (red line) with 95% confidence intervals (shaded area). The dashed line represents the quality benchmark of 12 nodes. **C** Comparative analysis of lymph node yield between laparoscopic and open approaches. In the recent era, the minimally invasive approach achieved a yield comparable to, and numerically superior to, open surgery (*P* = 0.067 in 2023), confirming non-inferiority in oncological clearance (Color figure online)

The surgical approach underwent a paradigm shift. The adoption of laparoscopic surgery surged from 0.2% in 2008 to 64.5% in 2023 (Fig. 2A). Despite the increasing complexity of the patient population, oncological quality metrics improved steadily. The mean total lymph node yield increased from 15.4 ± 7.4 in the open-predominant Era 1 to 16.9 ± 7.7 in the MIS-predominant Era 3 (*P* < 0.001, Fig. 2B). The proportion of patients achieving the benchmark yield of ≥ 12 nodes rose from 74.9% in Era 1 to 81.0% in Era 3 (Table 1). Circumferential resection margin (CRM) positivity also declined significantly over the study period, from 1.90% in Era 1 to 0.60% in Era 3 (*P* < 0.001). In the rectal cancer subgroup, CRM positivity decreased from 1.72 to 0.88% (*P* = 0.0006), providing additional evidence of maintained surgical oncological quality beyond lymph node yield.

Subgroup analysis in the final year of the study (2023) confirmed the non-inferiority of the minimally invasive approach. The mean lymph node yield in the laparoscopic group was 16.3 ± 6.8, which was comparable to, and numerically higher than, that of the open surgery group (15.6 ± 7.7; *P* = 0.067) (Fig. 2C). This suggests that the maturation of laparoscopic techniques has ensured rigorous oncological clearance even in routine practice.

### Survival outcomes

**Fig. 3:**
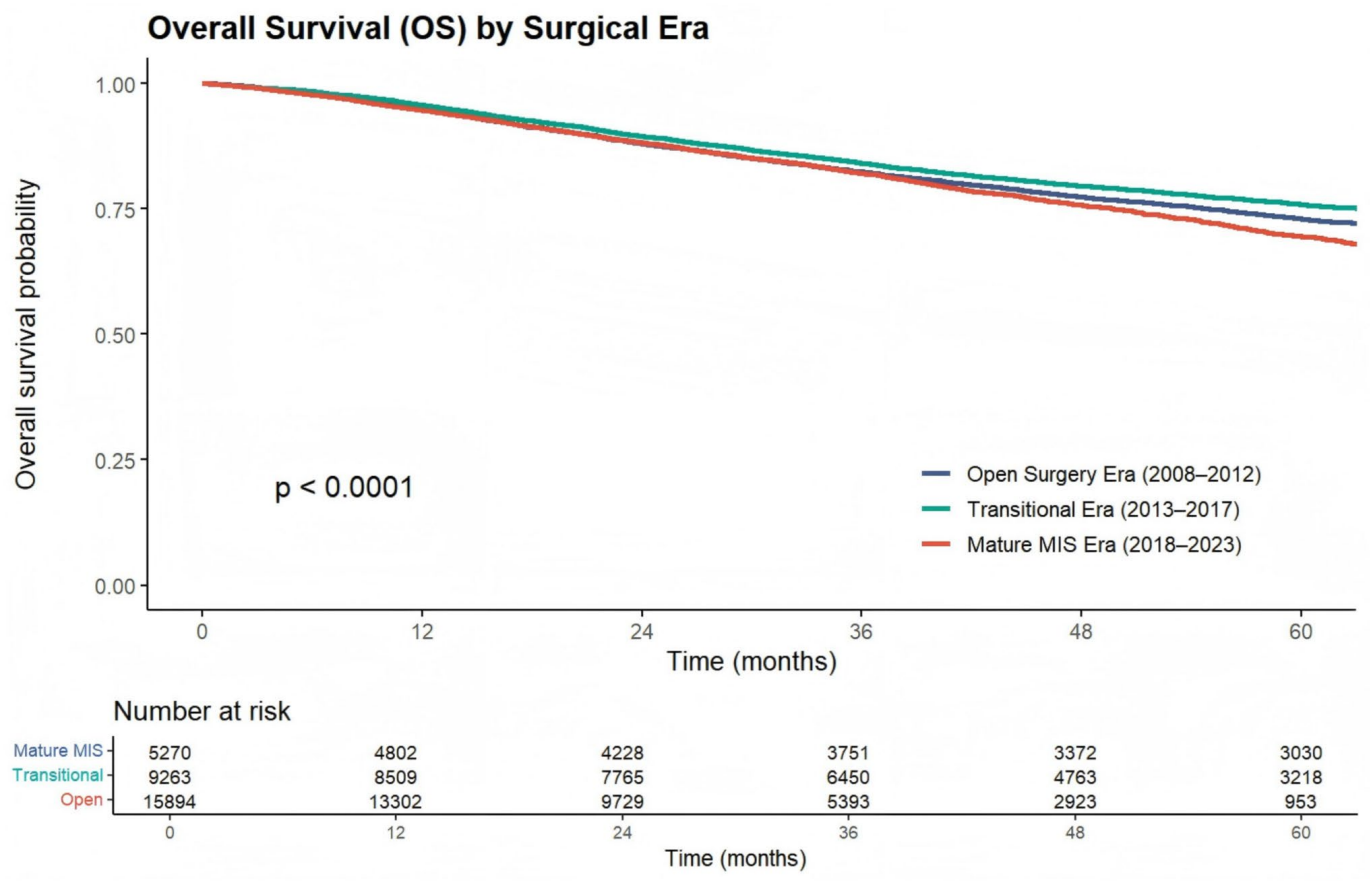
Long-term oncological outcomes stratified by surgical era (2008–2023). Kaplan–Meier estimates of 5-year overall survival (OS) are compared across three surgical eras. Era 1 (2008–2012, Blue) represents the baseline open-surgery period; Era 2 (2013–2017, Green) represents the transitional period; and Era 3 (2018–2023, Red) represents the mature MIS period. Despite the significantly older and more complex case-mix in Era 3, survival outcomes remained robust (69.5%) and comparable to the historical baseline, with the peak survival observed in the transitional Era 2 (75.9%, *P* < 0.001). The X-axis is truncated at 60 months to ensure comparable follow-up duration across cohorts (Color figure online)

Kaplan–Meier analysis demonstrated robust long-term outcomes despite the unfavorable shifts in patient age and stage (Fig. 3). The 5-year Overall Survival (OS) reached a peak of 75.9% during the transitional period (Era 2, 2013–2017). In the mature MIS era (Era 3, 2018–2023), the 5-year OS was 69.5%. While this figure was numerically lower than Era 2, it remained comparable to the historical baseline of Era 1 (73.0%). To account for the differences in age distribution and disease stage between eras, a multivariable Cox proportional hazards regression was performed (Table 2). After adjusting for age, sex, TNM stage, tumor location, and surgical approach, Era 3 showed no significant difference in mortality risk compared with Era 1 (HR = 1.049, 95% CI 0.976–1.128, *P* = 0.194), while Era 2 was associated with significantly improved survival (HR = 0.872, 95% CI 0.811–0.938, *P* < 0.001). A sensitivity analysis using a stratified Cox model (stratified by TNM stage) yielded consistent results (Era 3 vs Era 1: HR = 1.064, *P* = 0.095). These adjusted analyses confirm that the apparent decline in unadjusted OS in Era 3 was attributable to the unfavorable shifts in case-mix rather than a decline in surgical quality.

**Table 2:** Multivariable Cox regression for 5-year overall survival

Variable	HR	95% CI	*P* value
Era 2 (vs Era 1)	0.872	0.811–0.938	< 0.001
Era 3 (vs Era 1)	1.049	0.976–1.128	0.194
Age (per year increase)	1.021	1.018–1.023	< 0.001
Male sex	1.049	0.994–1.107	0.081
Stage II (vs Stage 0-I)	1.918	1.665–2.208	< 0.001
Stage III (vs Stage 0-I)	5.349	4.701–6.087	< 0.001
Stage IV (vs Stage 0-I)	15.083	13.210–17.223	< 0.001
Left colon (vs rectum)	0.872	0.816–0.933	< 0.001
Right colon (vs rectum)	1.087	1.020–1.157	0.010
Laparoscopic approach	0.972	0.913–1.035	0.373

### Sensitivity analyses

A sensitivity analysis excluding all 2,107 HGIN cases (*N* = 28,320) confirmed the robustness of the main findings. Demographic trends were virtually unchanged (EOCRC: 23.4% to 18.5%; late-onset: 29.9% to 37.8%), stage distribution shifts were preserved, and lymph node yield improvement persisted (Era 1: 15.6 ± 7.3 to Era 3: 17.0 ± 7.7). The adjusted Cox regression remained consistent (Era 3 vs Era 1: HR = 1.061, 95% CI 0.986–1.143, *P* = 0.114). Continuous time trend analyses using calendar year as a continuous variable confirmed a significant annual increase in lymph node yield (+ 0.138 nodes/year, *P* < 0.001) and a significant annual decline in the Stage 0–I proportion (− 0.50%/year, *P* < 0.001), independent of the era classification system (Supplementary Tables).

## Discussion

Our analysis of over 30,000 surgical cases spanning 16 years reveals a profound transformation in the landscape of colorectal cancer surgery. This study provides, to our knowledge, one of the largest single-center validations of the “endoscopic filtering effect” from a surgical perspective. Our data suggest that the modern surgical department is no longer a passive recipient of the general disease incidence but is increasingly becoming a specialized center for “refractory” demographics—specifically, older patients with locally advanced or complex disease—temporally associated with trends consistent with upstream diversion of early-stage cases.

### The paradox of EOCRC decline: a surgical perspective

A striking finding of our study is the steady decline in the proportion of surgically managed early-onset CRC (EOCRC), falling from 24.8% in 2008 to 14.6% in 2023. This trend stands in stark contrast to global epidemiological reports, which consistently highlight a rising incidence of EOCRC in the general population [3–5, 24]. We hypothesize that this “surgical paradox” represents a victory of screening rather than a divergence in disease biology. The concurrent, dramatic rise in the detection of HGIN (1.5% to 12.3%) and the contraction of surgical Stage 0–I cases (20.3% to 12.4%) strongly imply that younger patients—who are increasingly the target of opportunistic screening awareness—are being identified at the pre-invasive or early invasive stage. These patients are likely managed via endoscopic resection (e.g., ESD) and thus “filtered out” of the surgical statistics. Consequently, surgical EOCRC data should be interpreted with caution; in the era of advanced endoscopy, surgical series may increasingly reflect only “late-diagnosed” or “treatment-refractory” EOCRC, masking the broader success of early intervention [25–27].

We acknowledge that the “endoscopic filtering effect” remains a hypothesis supported indirectly by the observed temporal trends in surgical case-mix. Direct linkage to institutional endoscopy procedural volumes was not available, and thus a causal relationship cannot be established from surgical data alone. Nevertheless, the convergent evidence from multiple dimensions—demographic shifts, stage migration, tumor size trajectory, and HGIN detection rates—provides a coherent and plausible pattern consistent with this hypothesis.

### The "J-Shaped" tumor size and the de-early-stage phenomenon

The trajectory of mean tumor diameter—decreasing initially (2012–2014) and then rebounding after 2015—offers further morphological evidence of this filtering effect. The initial decline likely reflected the early dissemination of screening awareness. However, the subsequent rebound coincides with the widespread adoption of ESD as a standard treatment for large colorectal neoplasms (2–5 cm) in China [28, 29]. As "easy-to-resect" smaller lesions are diverted to endoscopy, the surgical cohort is progressively enriched with larger, bulkier, and technically more demanding tumors. This “de-early-stage” phenomenon necessitates that the modern colorectal surgeon must possess advanced technical proficiency to handle locally advanced disease, as the "simple" cases are disappearing from the operating theater [15, 30, 31].

### Defending quality in the face of demographic headwinds

The transition to Era 3 (2018–2023) presented a unique challenge: a “double hit” of an aging population (≥ 65 years: 39.4%) and a more advanced stage distribution. Historically, such a demographic shift would be expected to severely compromise survival outcomes [32, 33]. However, our data demonstrate that 5-year OS in Era 3 (69.5%) remained robust and comparable to the historical baseline of Era 1 (73.0%), with no evidence of the "learning curve" morbidity often associated with technical transitions.

This resilience is attributable to the maturation of minimally invasive surgery. Our analysis confirms that the laparoscopic approach has evolved from an experimental technique to a quality-enhancing standard. Not only did lymph node yield increase continuously (peaking at 16.9 in Era 3), but the laparoscopic group achieved a yield numerically superior to open surgery in the final year of the study (*P* = 0.067). Critically, multivariable Cox regression adjusting for age, sex, stage, tumor location, and surgical approach confirmed that Era 3 survival was not significantly different from the Era 1 baseline (HR = 1.049, *P* = 0.194), demonstrating that the apparent decline in unadjusted OS was attributable to case-mix changes rather than deteriorating surgical performance. Furthermore, CRM positivity declined from 1.90 to 0.60% overall and from 1.72 to 0.88% for rectal cancer (*P* = 0.0006), providing additional oncological quality evidence beyond lymph node yield. This suggests that the standardized view and meticulous dissection afforded by the laparoscopic platform have successfully offset the biological disadvantages of an aging, high-risk cohort.

### Limitations

This study has several limitations. First, as a single-center retrospective analysis conducted at a high-volume tertiary referral cancer center, our institution likely receives a disproportionate share of complex and locally advanced cases, potentially amplifying the observed trends toward increasingly advanced surgical case-mix. The demographic and staging shifts documented here may differ in magnitude at community hospitals or population-based settings. Population-based registries, such as the Shanghai Cancer Registry and the SEER database, would provide complementary perspectives to validate these findings. Additionally, referral patterns may have evolved over the study period as our institution’s catchment area expanded, which could independently contribute to the observed case-mix changes. Second, while we infer the "endoscopic filtering effect" from surgical data, we lacked direct linkage to our institution’s endoscopy database to quantify the exact volume of ESD procedures performed during the same period; thus, the filtering effect remains a hypothesis supported by indirect evidence. Third, survival analysis for the most recent years in Era 3 is limited by shorter median follow-up, although statistical adjustments were applied. Fourth, postoperative morbidity and conversion rates were not systematically captured in our institutional electronic medical record system during the early study years and could not be reliably reported across all three eras; surgical quality was therefore assessed through oncological clearance metrics (lymph node yield and CRM status). Fifth, the definition of surgical eras based on laparoscopic adoption could introduce circular reasoning when used to evaluate surgical performance; however, continuous time trend analyses using calendar year as a continuous variable confirmed all major findings independent of the era classification.

## Conclusion

Over the past 16 years, the profile of surgically managed CRC has shifted fundamentally from a broad-spectrum caseload to one enriched for older patients and locally advanced disease. These temporal shifts are consistent with, though not direct proof of, an upstream endoscopic filtering effect. In the face of this increasing complexity, the universal adoption of minimally invasive surgery has delivered sustained improvements in oncological quality and survival. Multivariable survival analysis confirmed that, after adjusting for confounders, oncological outcomes in the most recent era remained comparable to the historical baseline. Our findings suggest that modern surgical practice has successfully adapted to the challenges of a changing epidemiology.

## Supplementary Information

Below is the link to the electronic supplementary material.

Supplementary file1 (DOCX 30 KB)
