# Supplementary material for: Shifting paradigms in 30,427 surgical colorectal cancer cases (2008–2023): the impact of endoscopic screening on caseload complexity and the value of minimally invasive quality
Source: Surg Endosc. 2026 Apr 20;40(6):5101–10. doi: 10.1007/s00464-026-12818-1 (PMC13246532; doi:10.1007/s00464-026-12818-1)
Supplement: Supplementary file 1 — Supplementary file1 (DOCX 30 KB) [file 464_2026_12818_MOESM1_ESM.docx]

**Supplementary Table S1. Stratified Cox Proportional Hazards Model for 5-Year Overall Survival (Stratified by TNM Stage)**

| **Variable** | **HR** | **95% CI** | **P value** |
| --- | --- | --- | --- |
| Era 2 (vs Era 1) | 0.877 | 0.816–0.944 | <0.001 |
| Era 3 (vs Era 1) | 1.064 | 0.989–1.144 | 0.095 |
| Age (per year increase) | 1.021 | 1.018–1.023 | <0.001 |
| Male sex | 1.050 | 0.995–1.108 | 0.076 |
| Left colon (vs Rectum) | 0.873 | 0.817–0.934 | <0.001 |
| Right colon (vs Rectum) | 1.086 | 1.020–1.157 | 0.010 |
| Laparoscopic approach | 0.973 | 0.914–1.036 | 0.391 |

The proportional hazards assumption was tested using Schoenfeld residuals. Violations were detected for stage variables (P<0.05); therefore, a stratified Cox model was employed with stratification by TNM stage (0–IV). N = 28,383 after excluding cases with missing data.

**Supplementary Table S2. Multivariable Cox Regression with Age as a Categorical Variable**

| **Variable** | **HR** | **95% CI** | **P value** |
| --- | --- | --- | --- |
| Era 2 (vs Era 1) | 0.874 | 0.813–0.940 | <0.001 |
| Era 3 (vs Era 1) | 1.044 | 0.971–1.123 | 0.248 |
| Age 50–64 years (vs <50) | 1.058 | 0.982–1.140 | 0.138 |
| Age ≥65 years (vs <50) | 1.612 | 1.496–1.736 | <0.001 |
| Male sex | 1.060 | 1.005–1.119 | 0.032 |
| Stage II (vs Stage 0–I) | 1.923 | 1.669–2.214 | <0.001 |
| Stage III (vs Stage 0–I) | 5.309 | 4.665–6.043 | <0.001 |
| Stage IV (vs Stage 0–I) | 14.876 | 13.026–16.989 | <0.001 |
| Left colon (vs Rectum) | 0.871 | 0.815–0.932 | <0.001 |
| Right colon (vs Rectum) | 1.085 | 1.019–1.156 | 0.011 |
| Laparoscopic approach | 0.966 | 0.907–1.029 | 0.282 |

N = 28,531 after excluding cases with missing TNM stage or overall survival data.

**Supplementary Table S3. Stage-Stratified Survival Analysis by Era**

| **Stage** | **N** | **Era 2 vs Era 1 HR (95% CI)** | **P** | **Era 3 vs Era 1 HR (95% CI)** | **P** |
| --- | --- | --- | --- | --- | --- |
| Stage I | 4,387 | 0.751 (0.539–1.046) | 0.091 | 0.867 (0.605–1.243) | 0.438 |
| Stage II | 8,814 | 0.915 (0.754–1.111) | 0.371 | 1.412 (1.165–1.711) | <0.001 |
| Stage III | 11,285 | 0.889 (0.795–0.994) | 0.038 | 1.424 (1.280–1.585) | <0.001 |
| Stage IV | 3,595 | 0.849 (0.754–0.957) | 0.007 | 0.639 (0.564–0.724) | <0.001 |

Each stage-specific model was adjusted for age, sex, tumor location, and surgical approach. Era 1 served as the reference group.

**Supplementary Table S4. Circumferential Resection Margin (CRM) Status by Era**

**A. Overall Cohort**

| **Era** | **N** | **CRM Negative** | **CRM Positive** | **CRM+ Rate (%)** |
| --- | --- | --- | --- | --- |
| Era 1 (2008–2012) | 5,267 | 5,167 (98.1%) | 100 | 1.90 |
| Era 2 (2013–2017) | 9,260 | 9,148 (98.8%) | 112 | 1.21 |
| Era 3 (2018–2023) | 15,886 | 15,790 (99.4%) | 96 | 0.60 |

**B. Rectal Cancer Subgroup**

| **Era** | **N** | **CRM Negative** | **CRM Positive** | **CRM+ Rate (%)** |
| --- | --- | --- | --- | --- |
| Era 1 (2008–2012) | 2,729 | 2,682 (98.3%) | 47 | 1.72 |
| Era 2 (2013–2017) | 5,218 | 5,147 (98.6%) | 71 | 1.36 |
| Era 3 (2018–2023) | 7,992 | 7,922 (99.1%) | 70 | 0.88 |

Chi-square test for trend across eras (rectal cancer subgroup): P = 0.0006. CRM data were available for 30,437 patients (overall) and 15,939 patients (rectal subgroup).

**Supplementary Table S5. Sensitivity Analysis Excluding High-Grade Intraepithelial Neoplasia (HGIN): Demographics and Stage Distribution**

**A. Demographics (Excluding HGIN, N = 28,320)**

| **Variable** | **Era 1 (n=5,156)** | **Era 2 (n=9,058)** | **Era 3 (n=14,106)** |
| --- | --- | --- | --- |
| Age, years (Mean ± SD) | 58.1 ± 12.3 | 59.0 ± 11.7 | 59.7 ± 11.9 |
| <50 years, n (%) | 1,209 (23.4%) | 1,832 (20.2%) | 2,614 (18.5%) |
| ≥65 years, n (%) | 1,541 (29.9%) | 2,962 (32.7%) | 5,333 (37.8%) |

**B. Stage Distribution (Excluding HGIN)**

| **TNM Stage** | **Era 1 (n=5,059)** | **Era 2 (n=8,575)** | **Era 3 (n=13,285)** |
| --- | --- | --- | --- |
| Stage 0 | 96 (1.9%) | 87 (1.0%) | 20 (0.2%) |
| Stage I | 856 (16.9%) | 1,378 (16.1%) | 1,825 (13.7%) |
| Stage II | 1,416 (28.0%) | 2,633 (30.7%) | 4,284 (32.2%) |
| Stage III | 1,996 (39.5%) | 3,230 (37.7%) | 5,650 (42.5%) |
| Stage IV | 695 (13.7%) | 1,247 (14.5%) | 1,506 (11.3%) |

**C. 5-Year Overall Survival (Excluding HGIN)**

| **Era** | **N** | **5-Year OS (%)** | **Log-rank P** |
| --- | --- | --- | --- |
| Era 1 (2008–2012) | 5,107 | 72.7 |  |
| Era 2 (2013–2017) | 9,011 | 75.5 |  |
| Era 3 (2018–2023) | 14,092 | 68.4 | <0.001 |

**D. Lymph Node Yield (Excluding HGIN)**

| **Era** | **Mean ± SD** | **Adequate Yield (≥12 nodes)** |
| --- | --- | --- |
| Era 1 (2008–2012) | 15.6 ± 7.3 | 75.9% |
| Era 2 (2013–2017) | 16.3 ± 7.4 | 80.5% |
| Era 3 (2018–2023) | 17.0 ± 7.7 | 81.7% |

**Supplementary Table S6. Multivariable Cox Regression Excluding HGIN Cases**

| **Variable** | **HR** | **95% CI** | **P value** |
| --- | --- | --- | --- |
| Era 2 (vs Era 1) | 0.880 | 0.818–0.947 | <0.001 |
| Era 3 (vs Era 1) | 1.061 | 0.986–1.143 | 0.114 |
| Age (per year increase) | 1.021 | 1.018–1.023 | <0.001 |
| Male sex | 1.052 | 0.996–1.111 | 0.071 |
| Stage II (vs Stage 0–I) | 1.892 | 1.634–2.190 | <0.001 |
| Stage III (vs Stage 0–I) | 5.212 | 4.555–5.964 | <0.001 |
| Stage IV (vs Stage 0–I) | 15.180 | 13.223–17.427 | <0.001 |
| Left colon (vs Rectum) | 0.867 | 0.810–0.928 | <0.001 |
| Right colon (vs Rectum) | 1.086 | 1.019–1.158 | 0.012 |
| Laparoscopic approach | 0.978 | 0.917–1.043 | 0.496 |

N = 26,518 after excluding 2,107 HGIN cases and cases with missing data. Results were consistent with the primary analysis including HGIN (Table 2 of the main manuscript).

**Supplementary Table S7. Continuous Time Trend Analysis: Lymph Node Yield by Calendar Year**

| **Year** | **N** | **Mean LN Yield ± SD** | **Adequate Yield (≥12), %** |
| --- | --- | --- | --- |
| 2008 | 758 | 13.3 ± 6.7 | 64.4 |
| 2009 | 868 | 15.2 ± 8.0 | 72.4 |
| 2010 | 1,026 | 16.3 ± 8.2 | 76.3 |
| 2011 | 1,240 | 15.5 ± 6.4 | 76.3 |
| 2012 | 1,378 | 16.1 ± 7.4 | 78.4 |
| 2013 | 1,639 | 15.9 ± 7.2 | 77.7 |
| 2014 | 1,654 | 16.3 ± 7.0 | 80.3 |
| 2015 | 1,723 | 16.3 ± 7.1 | 80.3 |
| 2016 | 1,953 | 16.0 ± 7.7 | 78.7 |
| 2017 | 2,294 | 16.2 ± 8.0 | 79.0 |
| 2018 | 2,862 | 16.7 ± 7.8 | 80.3 |
| 2019 | 2,655 | 16.4 ± 6.9 | 81.0 |
| 2020 | 3,232 | 17.2 ± 7.8 | 82.1 |
| 2021 | 2,984 | 16.9 ± 7.8 | 80.8 |
| 2022 | 2,601 | 17.5 ± 8.2 | 81.8 |
| 2023 | 1,558 | 16.0 ± 7.2 | 79.5 |

Linear regression of lymph node yield on calendar year: slope = +0.138 nodes/year (P < 0.001). LN, lymph nodes.

**Supplementary Table S8. Continuous Time Trend Analysis: Stage Distribution and Cox Regression by Calendar Year**

**A. Stage 0–I Proportion by Calendar Year**

| **Year** | **N (staged)** | **Stage 0–I, n (%)** | **Stage III–IV, n (%)** |
| --- | --- | --- | --- |
| 2008 | 748 | 154 (20.6%) | 401 (53.6%) |
| 2009 | 859 | 174 (20.3%) | 433 (50.4%) |
| 2010 | 1,011 | 200 (19.8%) | 524 (51.8%) |
| 2011 | 1,214 | 251 (20.7%) | 625 (51.5%) |
| 2012 | 1,338 | 277 (20.7%) | 713 (53.3%) |
| 2013 | 1,566 | 320 (20.4%) | 777 (49.6%) |
| 2014 | 1,585 | 299 (18.9%) | 796 (50.2%) |
| 2015 | 1,623 | 292 (18.0%) | 823 (50.7%) |
| 2016 | 1,827 | 324 (17.7%) | 977 (53.5%) |
| 2017 | 2,159 | 357 (16.5%) | 1,146 (53.1%) |
| 2018 | 2,683 | 448 (16.7%) | 1,424 (53.1%) |
| 2019 | 2,462 | 361 (14.7%) | 1,337 (54.3%) |
| 2020 | 2,995 | 463 (15.5%) | 1,617 (54.0%) |
| 2021 | 2,730 | 443 (16.2%) | 1,374 (50.3%) |
| 2022 | 2,393 | 332 (13.9%) | 1,211 (50.6%) |
| 2023 | 1,449 | 196 (13.5%) | 740 (51.1%) |

Stage 0–I proportion trend: slope = −0.50%/year (P < 0.001, linear regression). Patients with missing TNM stage data were excluded from this analysis.

**B. Cox Regression with Calendar Year as Continuous Variable**

| **Variable** | **HR** | **95% CI** | **P value** |
| --- | --- | --- | --- |
| Calendar year (per year) | 1.017 | 1.009–1.024 | <0.001 |
| Age (per year increase) | 1.021 | 1.019–1.023 | <0.001 |
| Male sex | 1.049 | 0.994–1.107 | 0.080 |
| Stage II (vs Stage 0–I) | 1.919 | 1.666–2.210 | <0.001 |
| Stage III (vs Stage 0–I) | 5.348 | 4.699–6.085 | <0.001 |
| Stage IV (vs Stage 0–I) | 15.052 | 13.180–17.183 | <0.001 |
| Left colon (vs Rectum) | 0.874 | 0.817–0.935 | <0.001 |
| Right colon (vs Rectum) | 1.088 | 1.021–1.159 | 0.009 |
| Laparoscopic approach | 0.968 | 0.908–1.031 | 0.313 |

N = 28,531. The HR for calendar year represents the change in hazard per one-year increase, adjusted for all other covariates. This analysis provides an era-independent assessment of temporal trends in survival.
